# Oxidation of CO and Methanol on Pd-Ni Catalysts Supported on Different Chemically-Treated Carbon Nanofibers

**DOI:** 10.3390/nano6100187

**Published:** 2016-10-18

**Authors:** Juan Carlos Calderón, Miguel Rios Ráfales, María Jesús Nieto-Monge, Juan Ignacio Pardo, Rafael Moliner, María Jesús Lázaro

**Affiliations:** 1 Instituto de Carboquímica (CSIC), Miguel Luesma Castán 4, 50018 Zaragoza, Spain; jccalderon@icb.csic.es (J.C.C.); zoser1991@gmail.com (M.R.R.); mjnieto@icb.csic.es (M.J.N.-M.); rmoliner@icb.csic.es (R.M.); 2 Instituto Universitario de Investigación de Ingeniería de Aragón (I3A), Mariano Esquillor s/n, 50018 Zaragoza, Spain; jupardo@unizar.es

**Keywords:** Pd-Ni catalysts, carbon nanofibers, alkaline medium, CO oxidation, methanol oxidation, direct methanol fuel cells

## Abstract

In this work, palladium-nickel nanoparticles supported on carbon nanofibers were synthesized, with metal contents close to 25 wt % and Pd:Ni atomic ratios near to 1:2. These catalysts were previously studied in order to determine their activity toward the oxygen reduction reaction. Before the deposition of metals, the carbon nanofibers were chemically treated in order to generate oxygen and nitrogen groups on their surface. Transmission electron microscopy analysis (TEM) images revealed particle diameters between 3 and 4 nm, overcoming the sizes observed for the nanoparticles supported on carbon black (catalyst Pd-Ni CB 1:2). From the CO oxidation at different temperatures, the activation energy E_act_ for this reaction was determined. These values indicated a high tolerance of the catalysts toward the CO poisoning, especially in the case of the catalysts supported on the non-chemically treated carbon nanofibers. On the other hand, apparent activation energy E_ap_ for the methanol oxidation was also determined finding—as a rate determining step—the CO_ads_ diffusion to the OH_ads_ for the catalysts supported on carbon nanofibers. The results here presented showed that the surface functional groups only play a role in the obtaining of lower particle sizes, which is an important factor in the obtaining of low CO oxidation activation energies.

## 1. Introduction

Direct methanol fuel cells (DMFCs) have been proposed as alternative in the production of electricity for several mobile and stationary applications due to their high energy conversion efficiency, low work temperature, high power density, low pollution, quick start-up, and long lifetime [1]. However, the commercialization of these devices depends on the overcoming of some technical drawbacks as low activity of the anodic catalysts towards the methanol oxidation reaction (MOR) [2,3]. Moreover, the high cost of this technology is related to the employment of Pt-based catalysts in the anode, which results in a depleting of the Pt reserves, considering that the use of a suitable amount of this noble metal in the DMFCs is necessary to achieve adequate power [4].

A way to decrease the amount of Pt is the suitable dispersion of its nanoparticles on carbon supports [5,6,7,8]. Furthermore, design of non-noble metal catalysts with both high electrocatalytic activity and good stability has been suggested to replace Pt in the anodes [9]. In fact, some works showed the use of non-platinum anode catalysts in alkaline electrolytes, such as carbides [10], tungsten and molybdenum composites [11,12,13], manganese oxides [14,15], metal nitrides [16,17], and iridium oxides [18]. Pd-Ni alloys supported on carbon materials are another alternative. According to several works, the electrochemical oxidation of alcohols in alkaline medium on these alloys demonstrate outstanding performance [19,20,21] attributed to the key role of OH^−^ ions formed by Ni in the CO oxidation and thus, in the methanol oxidation [19]. These studies demonstrated that catalytic activity towards the oxidation of CO and methanol depends significantly on the alloy composition. For instance, Lim et al. [22] demonstrated that 1:1 Pd:Ni atomic ratio produces the best performance, in comparison with other different Pd:Ni atomic ratios. For this reaction, the electrochemical activity of Pd-Ni alloys has been also compared with that of Pd-Pt and Pd-Co alloyed materials, finding lower oxidation potentials [23]. Furthermore, some kinetic parameters for this have been determined [19,21], as well as the tolerance towards some carbonaceous intermediates formed during the methanol oxidation [24] and the study of the oxidation of other fuels as ethanol [25], ethylene glycol [26], formic acid [27], and allyl alcohol [20].

The role of carbon supports in the catalytic activity of anodes should also be evaluated in terms of their surface functional groups and structure. Regarding the surface oxygen groups, their presence improves the metal precursor impregnation during the synthesis procedures [28] and induces the electron transfer by means of their de-located free electrons [29]. Nitrogen groups can favor the obtaining of small particle diameters with high dispersion on the carbon support [30]. Regarding the structure, novel carbon materials have been designed in order to improve properties such as the diffusion of electroactive species, electrical conductivity, and stability. Some examples of these carbon supports are carbon nanotubes [31,32,33,34], hard carbon spherules [35], carbon microspheres [36,37], carbon aerogels and xerogels [38,39], and mesoporous carbons [40,41]. Additionally, carbon nanofibers (CNFs) have demonstrated excellent textural properties and high electrical conductivities, making them suitable as supports for anode catalysts [42,43]. In this work, Pd-Ni nanoparticles supported on chemically treated carbon nanofibers have been tested for the oxidation of CO and methanol in order to suggest them as possible anodes in DMFCs. These catalysts displayed a high activity toward the oxygen reduction reaction, suggesting that they can be a good alternative to replace Pt in the electrodes of alkaline direct methanol fuel cells [44].

## 2. Results

### 2.1. Chemical and Physical Properties of Carbon Supports

**Figure 1:**
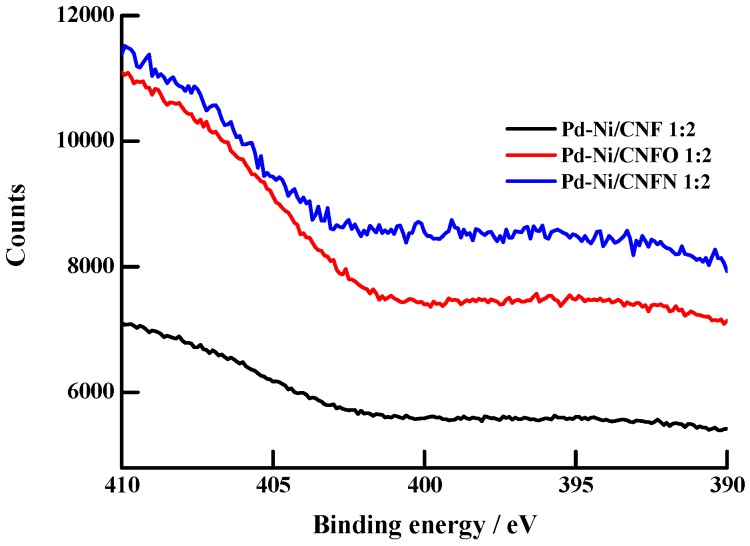
N 1 s X-ray photoelectron spectroscopy (XPS) spectra for the synthesized Pd-Ni catalysts supported on carbon nanofibers.

Table 1 shows some textural and chemical properties of carbon nanofibers and carbon black supports. Chemical treatment increased the surface area and pore volume of CNFs, being the N-modified carbon nanofibers which displayed the noticeable changes in these properties. Nonetheless, carbon black exhibited the biggest values for these parameters. Reaction between ethylenediamine and the carbon nanofibers with surface oxygen groups (CNFO) induced the formation of surface nitrogen groups, considering the nitrogen content found in the CNFO treated with ethylenediamine (CNFN). The formation of these nitrogen groups is a consequence of the reaction between the acidic oxygen groups on CNFO with a basic molecule such as ethylenediamine [45]. This result was corroborated by means of the resolved X-ray photoelectron spectroscopy (XPS) spectra for the 1 s transitions of N, located toward 397.9 eV and showed in Figure 1 [46]. The spectra demonstrated that the presence of nitrogen in CNFN is bigger than that of the CNFO and CNF supports, corroborating the presence of surface nitrogen groups in this carbon material. CNFO also showed a higher N signal than that of CNF, attributed to the insertion of some nitrogen after the treatment of the CNF material with concentrated nitric acid [47]. Table 1 also showed that mesopore volumes of carbon nanofibers were increased after the chemical treatments of the nanofibers, an important result considering the key role of these mesopores in the diffusion of electroactive species toward the catalytic nanoparticles [44].

**Figure 2:**
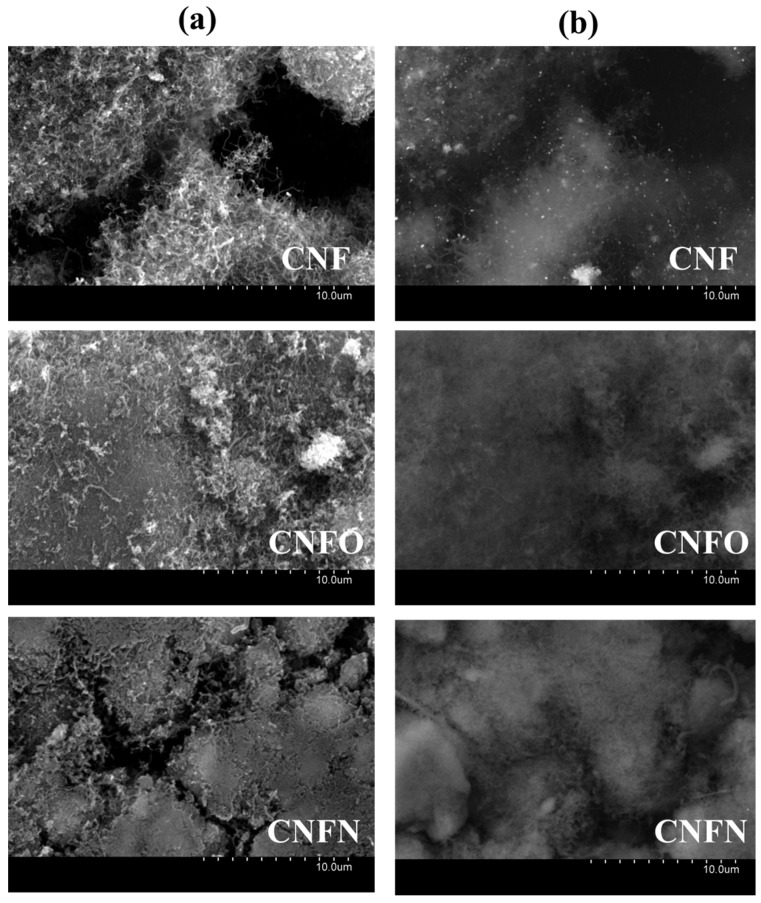
(**a**) Scanning electron microscopy (SEM) images of the carbon nanofibers used as support for the synthesized catalysts; (**b**) retrodispersed images of the images presented in (**a**).

Figure 2 presents the scanning electron microscopy (SEM) images of the carbon nanofibers before supporting the Pd-Ni nanoparticles. In the column (a) of these images it is possible to observe a partial loss and a compaction process of the CNF structure after the chemical treatments, as reported in previous works [43,48]. Column (b) displayed the retrodispersed images of the SEM micrographs, with the bright phase representing the presence of Ni in the nanofibers. This metal was especially evident in the case of the carbon nanofibers without any chemical treatment, indicating that some Ni dregs coming from the catalyst employed during the growth of the nanofibers remain in the final material. After chemical treatment with HNO_3_, the Ni is totally eliminated, as can be seen in the retrodispersed image of CNFO and CNFN. This result indicates that acid treatment was useful to eliminate the content of residual Ni [43,49].

**Figure 3:**
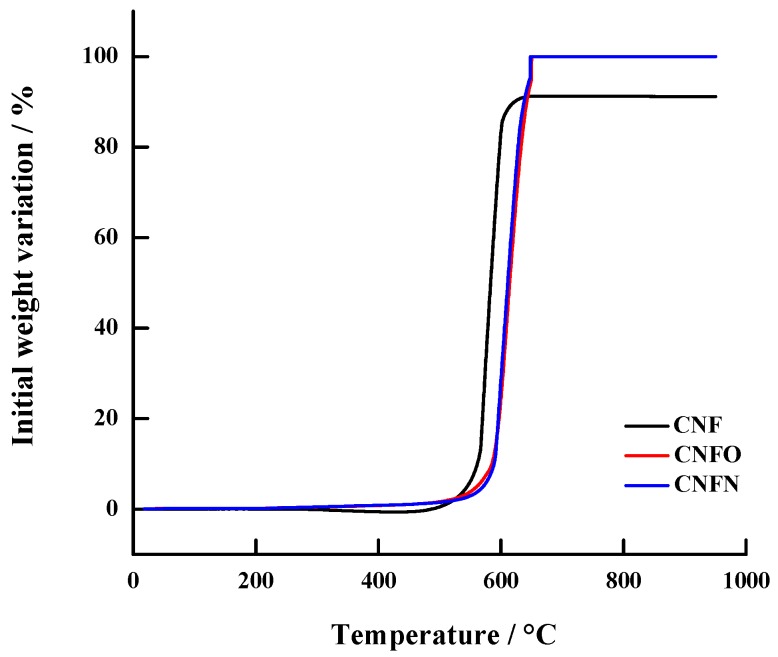
Thermogravimetric analysis (TGA) of the carbon nanofibers used as support of the Pd-Ni catalysts.

The behavior described above can be verified by means of the weight variation profiles obtained by thermogravimetric analysis (TGA), which are depicted in Figure 3. These profiles show that CNFO and CNFN achieved a total variation of the initial weight at 650 °C approximately, as a consequence of the total decomposition of the carbon nanofibers with the increase of the temperature. However, in the case of CNF, this decomposition did not achieve 100%, due to the presence of the residual Ni in the nanostructure. The decomposition in CNF was close to 91%, suggesting a 9% of residual Ni in CNF.

### 2.2. Physical Characterization of Catalysts

**Figure 4:**
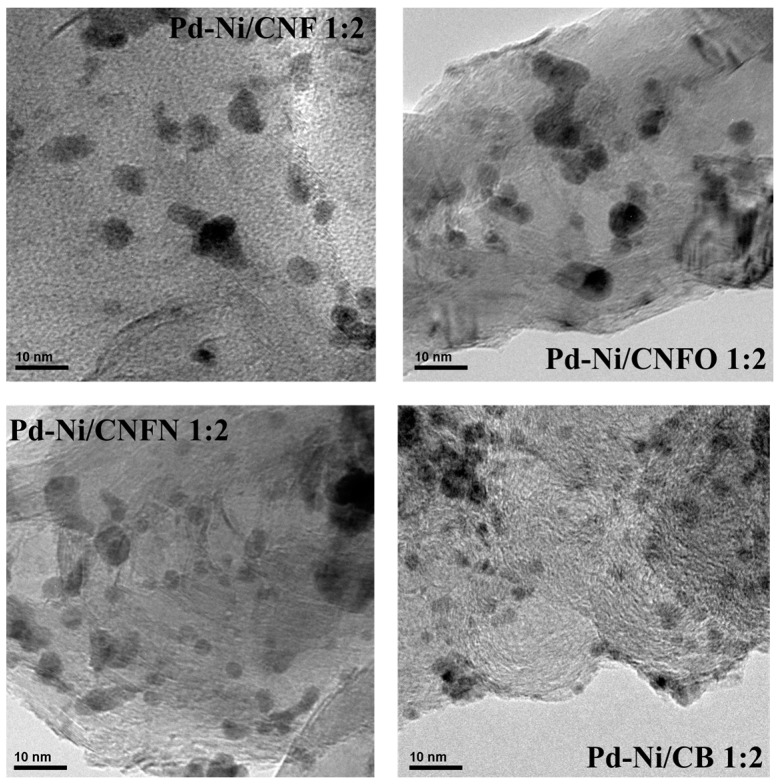
Transmission electron microscopy analysis (TEM) images of the synthesized Pd-Ni catalysts.

X-ray diffraction (XRD) characterization of the catalysts has been widely described in [44], showing a low alloying degree between Pd and Ni, due to the low temperatures employed during the synthesis of the catalysts [50,51]. Composition and particle size of synthesized nanoparticles are reported in Table 2. Energy dispersive X-ray (EDX) analysis showed metal contents close to those planned during the preparation (25 wt %), as well as the Pd:Ni atomic ratios were near to 1:2. Transmission electron microscopy analysis (TEM) images exhibited high nanoparticle dispersion on carbon nanofibers, with only few aggregates (see Figure 4), suggesting that carbon nanofibers promote the obtaining of well-dispersed nanoparticles. Regarding the particle size, diameters between 3.4 and 4.1 nm were observed for the catalysts supported on CNFs, overcoming the value observed for the catalyst Pd-Ni/CB 1:2, which is supported on carbon black [44].

**Figure 5:**
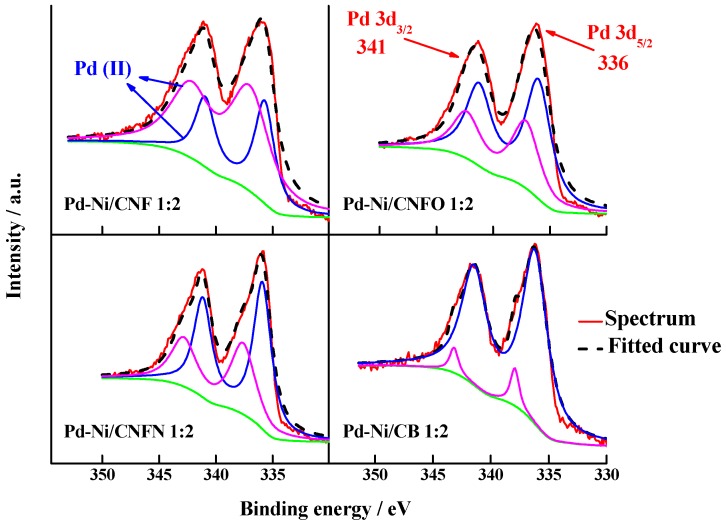
Pd 3d XPS spectra for the synthesized Pd-Ni catalysts supported on carbon nanofibers and carbon black.

The catalysts were analyzed by XPS to determine the surface electronic states of Pd and Ni. Figure 5 shows the deconvolution of the binding energy spectra for Pd 3d, which is composed by two doublet peaks related to the transitions occurring in the 3d_5/2_ and 3d_3/2_ orbitals. Table 3 displays the assignment and relative abundance for the oxidation states associated with these transitions. They are located in a small range between 336 and 341 eV, corresponding to the Pd (II) oxidation state [46]. This result indicates that carbon nanofibers favor the formation of Pd oxides with low oxidation states, whereas the carbon black is able to form both Pd (II) and Pd (IV), the last one in a low proportion. Surface Pd:Ni atomic ratio was also determined, finding an increase in the Ni content in comparison with the values observed for the bulk determined by EDS (see Table 2). The catalyst with the highest content of Ni was that supported on carbon black (92%), while in the case of the catalysts supported on carbon nanofibers, the content of Ni was in the range of 79%–85% with respect to Pd. This result indicates that the nickel observed in the retrodispersed SEM images and determined in CNF by TGA is encapsulated in the nanofiber structure. In agreement with this result, we can observe the increase in the surface Ni atomic content for the catalysts supported on carbon nanofibers with surface functional groups. This change was between 79% in Pd-Ni/CNF 1:2 and 85 and 84% in Pd-Ni/CNFO 1:2 and Pd-Ni/CNFN 1:2, respectively, suggesting a role of surface functional groups in the increase of the surface Ni content.

**Figure 6:**
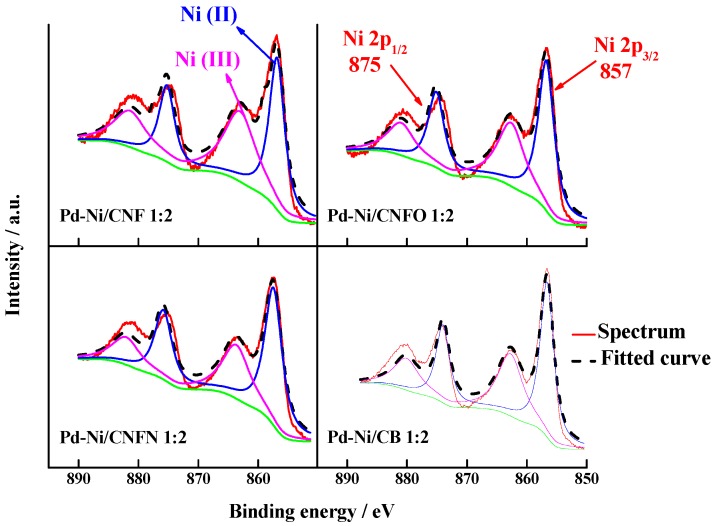
Ni 2p XPS spectra for the synthesized Pd-Ni catalysts supported on carbon nanofibers and carbon black.

In the case of the nickel, the transitions correspond to the 3p orbitals and the XPS signals are shown in Figure 6. The signals are located at 857 and 875 eV and correspond to the 2p_3/2_ and 2p_1/2_ transitions of Ni(II) and Ni(III) oxidation states, respectively [46]. There is a slight difference between the relative abundance for these oxidation states, with Ni(II) having the bigger concentration for all the studied catalysts (see Table 3). The catalysts supported on CNFO and CNFN displayed a higher content of Ni(II), suggesting that these groups promote the formation of Ni(II).

### 2.3. Electrochemical Activity Towards CO and Methanol

**Figure 7:**
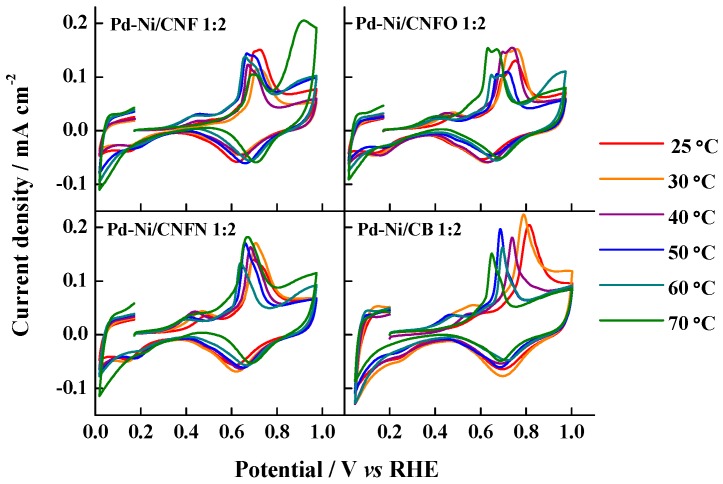
CO stripping voltammograms for the synthesized Pd-Ni catalysts at the temperature range (20–70 °C). Support electrolyte: 0.1 M KOH. Scan rate: 20 mV·s^−1^. E_ad_ = 0.2 V vs. reference hydrogen electrode (RHE).

Figure 7 shows the CO strippings performed on the different catalysts between 25 and 70 °C. In all the cases, the increase of temperature induced a shift in the CO oxidation peak toward more negative potential values—according to the Arrhenius law—as the catalyst supported on carbon black (CB) exhibited the most evident shift. Moreover, several CO oxidation pre-peaks were observed, as well as some peaks displaying doublet signals, indicating different surface compositions, which can modify the CO adsorption energy on the catalyst surface. This behavior has also been observed for the CO oxidation on heat-treated Pt-Ru catalysts supported on carbon nanofibers [52]. Furthermore, an increase in the current densities was observed once the CO oxidation peak potential is defined, particularly at the highest temperatures, possibly caused by the surface modification of the catalysts, generating heterogeneous zones where CO adsorption is promoted at different energy. This fact was very noticeable in the case of the materials Pd-Ni/CNF 1:2 and Pd-Ni/CNFN 1:2, at 60 and 70 °C; for instance, the catalyst Pd-Ni/CNF 1:2 at 70 °C required more positive potentials for the total oxidation of CO_ads_ monolayer, this fact being another proof of the different surface composition of the catalysts and their modification after the temperature increase. These results also suggest that catalysts supported on carbon nanofibers are less poisoned between 20 and 50 °C.

**Figure 8:**
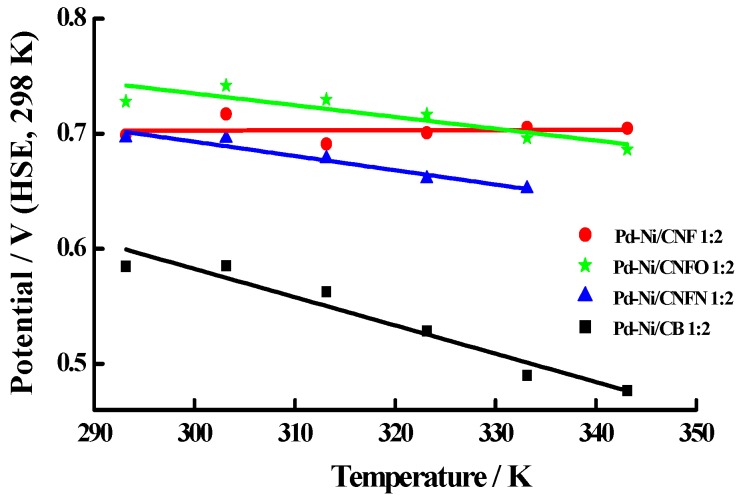
CO oxidation peak potential vs. temperature for the synthesized Pd-Ni catalysts.

The CO oxidation peak potential values were employed to determine the activation energy E_act_ for this reaction, following the procedure reported by Rincón et al. [53]. In this procedure, potential values are plotted against temperature, with the Y-axis intercept indicating the E_act_. Figure 8 presents the linear trends for this representation and Table 4 displays the E_act_ values, which were lower than those reported by Calderón et al. [54] for the oxidation of CO on some Pt-Ru catalysts supported on carbon nanofibers and the commercial catalyst, Pt-Ru E-TEK. A remarkable fact was that lower E_act_ values were observed for the materials supported on carbon nanofibers, in comparison with the high value observed for the Pd-Ni catalyst supported on carbon black (Pd-Ni/CB 1:2). The lowest E_act_ was displayed by the catalyst supported on the non-chemical treated carbon nanofibers (Pd-Ni/CNF 1:2, 68 kJ·mol^−1^), whereas the presence of surface groups on the carbon support increased the E_act_ values (101 kJ·mol^−1^ for PdNi/CNFO 1:2 and 103 kJ·mol^−1^ Pd-Ni/CNFN 1:2). This result does not agree with that presented by Calderón et al. related with the oxidation of carbon monoxide on Pd-Ni nanoparticles supported on carbon blacks [50], which was promoted on the catalysts supported on the carbon blacks with surface oxygen and nitrogen groups. In this sense, it must be considered that structure, morphology, and conductivity of carbon supports can modify the activity of catalysts [55]. In the case of carbon nanofibers, their better conductivity—which is attributed to the presence of graphite planes [42,43]—probably enhanced the catalytic activity of Pd-Ni/CNF 1:2. However, chemical treatments applied to create oxygen and nitrogen groups could induce a loss of crystalline mesoporous structure (see Figure 2), thus decreasing the catalytic activity of Pd-Ni/CNFO and Pd-Ni/CNFN. In the case of carbon blacks, this effect is not too crucial, since carbon black is basically amorphous, even after the chemical treatments, suggesting that these surface functional groups facilitate the oxidation of CO_ads_ [50]. In all the cases, the tolerance of the synthesized Pd-Ni catalysts is similar to those commonly reported in literature [53,54] and reasonably in agreement with those calculated by molecular orbital theory (135 kJ·mol^−1^) for the E_act_ of the reaction between Pt-OH_ad_ and Pt-CO_ad_ at 1 V [56].

**Figure 9:**
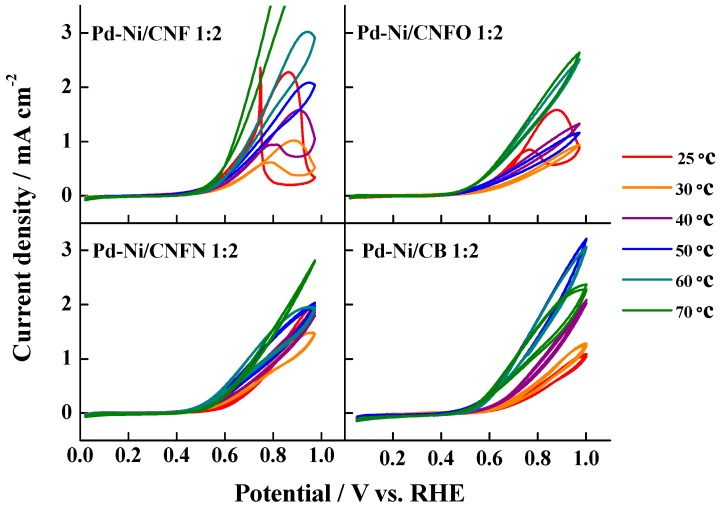
Methanol oxidation voltammograms for the synthesized Pd-Ni catalysts at the temperature range (20–70 °C). Support electrolyte: 0.1 M KOH. Scan rate: 20 mV·s^−1^. Methanol concentration: 1.0 M.

The methanol oxidation on the synthesized catalysts was also studied and the results are shown in Figure 9. In all the range of temperatures, the catalysts supported on the carbon nanofibers without any chemical treatment exhibited the highest current densities, suggesting an increased catalytic effect caused by the residual nickel present in the carbon nanofibers, even though this Ni is encapsulated in the carbon nanofiber structure. Furthermore, it means that there is no role of the surface oxygen or nitrogen groups in this reaction. The Pd-Ni catalysts supported on the chemically-modified carbon nanofibers displayed similar current densities to those of the catalyst supported on carbon black, indicating no effects associated to the fibers structure on this reaction. However, a hysteresis effect was detected for the catalysts Pd-Ni/CNF 1:2 and Pd-Ni/CNFO 1:2, which was not seen in Pd-Ni/CB 1:2. This hysteresis is explained from the oxidation of some species formed during the forward scan, which are re-oxidized in the backward scan [51,57,58]. Moreover, the catalysts synthesized on carbon nanofibers presented a continuous enhancement of the current densities with the increase of temperature, in comparison with the behavior observed for Pd-Ni/CB 1:2, which exhibited their highest current densities at 50 °C and a current density drop at 60 and 70 °C. This fact suggests the formation of a minor passivizing oxides layer with the increase of temperature for the metal nanoparticles supported on carbon nanofibers, making them more active. It is also possible that high conductivity of this support can compensate the effect of the formation of the Pd oxides layer.

**Figure 10:**
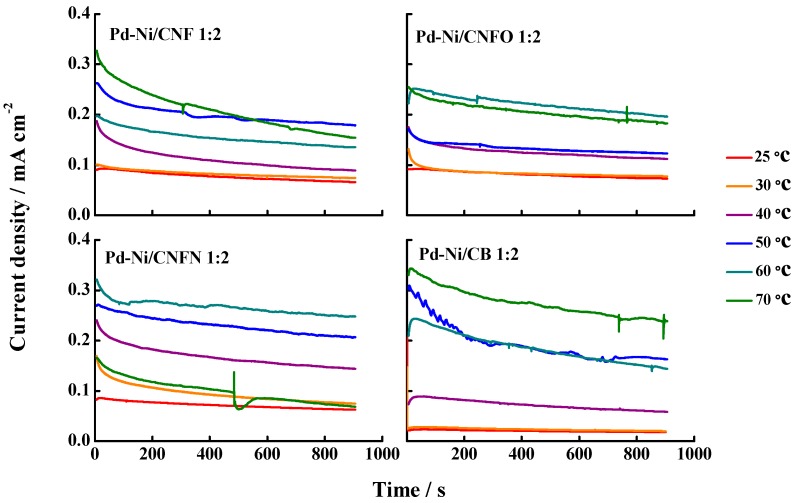
Selected current transients for the synthesized Pd-Ni catalysts at the temperature range (20–70 °C). Support electrolyte: 0.1 M KOH. Scan rate: 20 mV·s^−1^. Methanol concentration: 1.0 M. Applied potential: 0.6 V vs. RHE.

Current density vs. time curves (Figure 10) were recorded in order to evaluate the methanol oxidation at 0.6 V vs. RHE, a typical operation potential in the DMFCs. The values for these stationary methanol oxidation current densities were similar to those observed during the cyclic voltammetry experiments, demonstrating that there is no effect of the surface groups on the methanol oxidation. The increase of temperature up to 60 and 70 °C induced a drop in the current densities in some catalysts, caused by either bubbles and/or movement of methanol solution.

**Figure 11:**
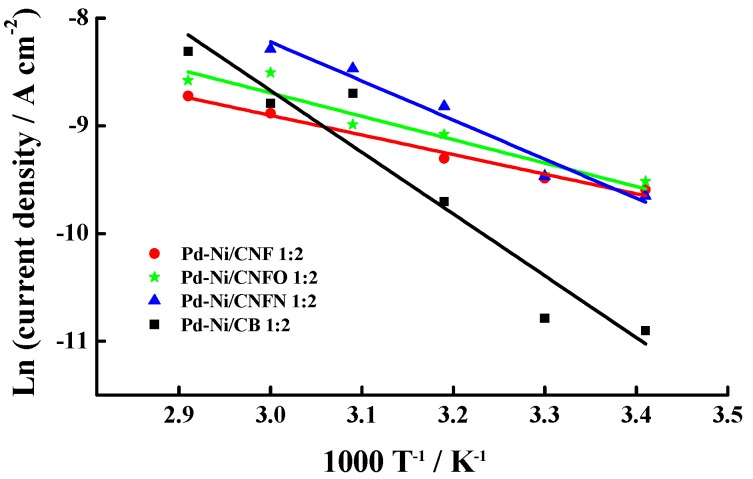
Arrhenius plots for the synthesized Pd-Ni catalysts.

From the stationary current density values, apparent activation energies E_ap_ for the methanol oxidation on the catalysts were determined, following the procedure suggested by Cohen et al. [59], which allows us to determine this parameter from the slopes of the Arrhenius plots obtained for each catalyst. Figure 11 shows the Arrhenius plots and Table 5 reports the values of the E_ap_, which were lower than 30 kJ·mol^−1^. Bearing in mind that methanol oxidation follows the bi-functional mechanism [60]:

(1) $$CH_{3}OH⇌CH_{3}OH{\;}_{ads}$$

(2) $$CH_{3}OH{\;}_{ads}\to CO_{ad}+4H^{+}+4e^{-}$$

(3) $$H_{2}O\to OH_{ad}+H^{+}+e^{-}$$

(4) $$CO_{ad}+OH_{ad}\to CO_{2}+H^{+}+e^{-}$$

It is possible to suggest which are the rate determining steps (*rds*) for this reaction, according to the determined E_ap_ values. In this sense, the values lower than 30 kJ·mol^−1^ indicate that CO surface diffusion toward the OH_ads_ formed on the Ni oxides is the *rds*, as in the case of the catalysts supported on carbon nanofibers [61,62]. On the other hand, the catalyst supported on carbon black presented the highest E_ap_ value (47.9 kJ·mol^−1^), suggesting methanol deprotonating as *rds* when the methanol oxidation is performed on Pd-Ni/CB 1:2. Bear in mind that the catalyst Pd-Ni/CB 1:2 has the highest surface Ni content, as described previously from the XPS analysis (see Table 3), but the *rds* on this catalyst was the methanol deprotoning instead of the CO surface diffusion toward the OH_ads_. Considering this, it is possible to suggest a higher stability of the OH_ads_ formed on the surface Ni present in the catalysts supported on carbon nanofibers, which could be induced by the structure of this carbon support, which allows them to address the reaction mechanism through the CO_ads_ diffusion toward the OH_ads_ as the rate determining step for this reaction.

## 3. Discussion

The results presented in this work have shown that it is possible to use Pd-Ni catalysts supported on carbon nanofibers as an alternative to replace Pt as catalytic phase in the anode of direct methanol fuel cells. This statement is justified in the good morphological and catalytic properties observed for these materials. It seems that carbon nanofibers promoted the formation of well-dispersed nanoparticles without agglomerates. Nevertheless, the particle size was bigger than those reported in a recent work, related with the synthesis of Pd-Ni catalysts supported on chemically modified carbon blacks [50]. In this work, the authors reported particle sizes lower than 3 nm for catalysts supported on carbon blacks, whereas the CNF-supported catalysts showed in the present manuscript overcame this value (sizes close to 4 nm). Although this result would imply a decrease in the surface metallic area, the electrochemical characterization has demonstrated an increase in the electrochemical activity. Indeed, high tolerance to the CO poisoning was appreciated from the low E_act_ values determined when this reaction is performed on the CNF-supported catalysts, possibly indicating an increase in the amount of active crystallographic planes associated with the high particle sizes. Another beneficial effect of particle size could be related to the high activity of the catalysts towards the methanol electrochemical oxidation, considering the high current densities observed for the materials supported on carbon nanofibers, especially at low temperatures, in comparison with those observed for the catalyst supported on carbon black (see Figure 9). This result could be explained from the high tolerance toward the CO poisoning, which is formed during the methanol oxidation, even bearing in mind that the surface diffusion of CO_ads_ toward the OH_ads_ was the rate determining step for this reaction, according to the E_ap_ values determined from the Arrhenius plot and the methanol oxidation stationary current densities at 0.6 V.

The effect of surface functional groups present on carbon nanofibers in the catalytic activity of the synthesized materials was related with the decrease in the particle size of the nanoparticles, a fact that probably affected the tolerance of the materials toward the CO poisoning, as described above. Nevertheless, in the case of the methanol oxidations, it seems that the good properties of carbon nanofibers—such as high conductivity and mesoporous content—play a key role during the methanol oxidation, in terms of the re-oxidation of carbonaceous species formed during the forward potential scan. Moreover, the structure of these CNFs determined the CO_ads_ diffusion toward the OH_ads_ as the *rds*, whereas this step corresponded to the methanol deprotoning in the case of the carbon black-supported catalyst.

Bearing in mind that the purpose of this work is to suggest an alternative to replace the platinum in the anodes of DMFCs, it is important to compare the results presented in this work with some reported for commercial Pt catalysts supported on carbon blacks in terms of their activity toward the methanol electrochemical oxidation. Recently, a 20 wt % Pt catalyst supported on Vulcan XC-72R was studied in this reaction in alkaline media (1.0 M NaOH + 1.0 M CH_3_OH) and displayed maximum current densities close to 15 mA·cm^−2^ [63], this is value five times bigger than that of Pd-Ni/CNF. It must be considered that this catalyst presented the highest performance within the group of catalysts supported on carbon nanofibers. In other work, a commercial catalyst Pt/C E-TEK exhibited current densities near to 0.1 A·mg^−1^_metal_ for the oxidation of a 0.5 M methanol solution in 0.5 M KOH [64]. The Pd-Ni catalysts here studied showed mass activities close to 0.085 A·mg^−1^_metal_, a relatively close value to that exhibited for the reported commercial catalyst. From the revision of these reported values, it is possible to suggest that our Pd-Ni catalysts supported on carbon nanofibers have an acceptable performance—although there is potential for improvement—in order to overcome the current densities displayed for the commercial Pt/C catalysts.

Moreover, the results obtained in this work can be compared with those obtained for some Pd-based catalysts supported on other kind of support, as TiO_2_ nanowires and nanotubes. The use of these non-carbonaceous supports has been promoted considering the high resistance to corrosion displayed by these materials when they are present in the electrodes of DMFCs [65,66,67]. For instance, Liang and co-workers supported Pd nanoparticles on a TiO_2_-carbon composite with a cross-linked-nanobelts structure [68]. This material displayed methanol oxidation current densities close to 4.5 mA·cm^−2^, whereas the Pd nanoparticles supported on TiO_2_ and activated carbon exhibited current densities near to 2.5 and 2.0 mA·cm^−2^, respectively. This improved activity was attributed to a better diffusion of methanol through the TiO_2_-C composite and its enhanced conductivity, in comparison with that of the TiO_2_ support. In other work, Hosseini et al. tested the activity of Pd nanoparticles supported on a TiO_2_ nanotubes-titanium composite [69], finding maximum current densities of 23 mA·cm^−2^ in a 0.5 M methanol solution prepared in alkaline media (1.0 M KOH), which were attributed to the improved diffusion of methanol through the support toward the Pd nanoparticles and the enhanced electrochemical charge transfer in the activation step for the methanol oxidation. The currents detected for these authors are higher than those observed in our work, although it must be considered that we employed a high methanol concentration (1.0 M Methanol), which probably induced a major production of intermediates during the methanol oxidation, poisoning the catalytic sites of the electrode. Ju and co-workers deposited Pd-Ni nanoparticles on TiO_2_ nanotubes to perform the oxidation of methanol in acid media [70]. They found maximum currents of 12 mA without any normalization, which allows us to conclude that, in this case, these currents can be comparable with those of our work. The authors compared the Pd-Ni catalyst with a commercial PtRu/C, finding enhanced currents, as a consequence of the porous structure and large surface area that improved the dispersion of the catalytic nanoparticles.

## 4. Materials and Methods

### 4.1. Carbon Supports

Catalytic thermal decomposition of methane was performed to grow the carbon nanofibers (CNFs) [71], employing a Ni:Cu:Al catalyst (atomic ratio = 78:6:16, Instituto de Carboquímica-CSIC, Zaragoza, Spain) and passing a flux of methane (Air Liquide, Zaragoza, Spain) at 700 °C during 10 h on this catalyst [72]. Subsequent, CNFs were treated in HNO_3_ 65% (v/v) (Sigma-Aldrich S.L., Madrid, Spain) for 2 h at 110 °C to form surface oxygenated groups and remove the metals from the catalyst (Ni, Al, and Cu, Instituto de Carboquímica-CSIC, Zaragoza, Spain) [73]. This new carbon support was labeled as CNFO. The last chemically-modified carbon nanofibers were the nitrogen-modified carbon nanofibers (CNFN), which were obtained by mixing CNFO with ethylenediamine (Sigma-Aldrich S.L., Madrid, Spain), in a 10:6 molar ratio at room temperature during 24 h. As mentioned in Section 2.1, this reaction promotes the formation of surface nitrogen groups, due to the acidic properties of surface oxygen groups present in CNFO and the basic functional groups of ethylenediamine [45]. After, the nanofibers were washed to pH 7.0 and dried at 85 °C for 24 h. Carbon black (Cabot^®^, Lillebone, France) was also employed for preparing a Pd:Ni 1:2 catalyst, in order to compare it with those supported on carbon nanofibers. The chemical composition of the carbon nanofibers was determined by means of an elemental analyzer Thermo Flash 1112 (Thermo Scientific, Delft, The Netherlands), taking the sample of the nanofibers and burning it at 1000 °C in an oxygen (Air Liquide, Zaragoza, Spain) atmosphere. The different gases were transported by a Helium flow and then analyzed in a thermal conductivity detector. For the thermogravimetric analysis (TGA), a SETARAM Setsys Evolution thermobalance (SETARAM Instruments, Cailure, France) was employed, which was operated between 19 and 980 °C in an O_2_ atmosphere.

### 4.2. Preparation of Pd-Ni Catalysts

A detailed description of the catalysts is performed in [44]. Briefly, carbon materials were dispersed in ultra-pure water by sonication and magnetic stirring. Then, the precursor salts solution (Na_2_PdCl_4_, 98 wt %, NiCl_2_, 99.999 wt %, Sigma-Aldrich S.L., Madrid, Spain) was added to the dispersion. Later, pH was adjusted to 5.0 with a concentrated NaOH solution (98%, Panreac, Castellar del Vallès, Spain) and kept in stirring during 12 h. After, a 26.4 mM sodium borohydride solution (99%, Sigma-Aldrich S.L., Madrid, Spain) was slowly added under sonication. The mixture was kept under magnetic stirring during 12 h, before filtering, washing, and drying the product at 60 °C. The as-synthesized catalysts were labeled in agreement with the used carbon support and the Pd:Ni atomic ratio. The names of these materials were: Pd-Ni/CNF 1:2, Pd-Ni/CNFO 1:2, Pd-Ni/CNFN 1:2, and Pd-Ni/CB 1:2.

### 4.3. Physical Characterization

Pd-Ni atomic ratios and metal content of the materials were determined by energy dispersive X ray analysis (EDX). A scanning electron microscope Hitachi S-3400 N (Singapore) was used, which was coupled to a Röntec XFlash analyzer operating at 15 keV. This microscope worked with a Si(Li) detector and a Be window. The carbon nanofibers and catalyst powders were put in a steel sampler, and five points in different regions of the deposit were tested, at 5000 magnification increment units. The results here reported correspond to the average of these measurements.

Particles size distribution and dispersion were determined by transmission electron microscopy analysis (TEM). A transmission electron microscope (200 kV JEOL-2000 FXII, Peabody, MS, USA) was used, dropping an ethanol suspension of the catalysts on a carbon grid. Images were obtained with a MultiScan CCD (Gatan 694) camera.

X-ray photoelectron spectroscopy (XPS) analysis was made by means of a ESCAPlus Omicron spectrometer (Scientia Omicron, Taunusstein, Germany) equipped with a concentric hemispherical analyzer and seven channeltron detectors and an Al/Mg monochromated X-ray source (K_α_ = 1253.6 eV)—this source operated at 15kV and 15 mA. The XPS spectra were recorded at pressures lower than 8 × 10^−9^ mbar. They were processed using the XPSPEAK 4.1^®^ software, fitting the experimental data with Gaussian-Lorentzian curves to identify the chemical states of Pd and Ni and the surface composition of the Pd-Ni nanoparticles [74].

### 4.4. Electrochemical Characterization

A three electrode cell connected to an AUTOLAB NS 85630 modular equipment (Metrohm, Herisau, Switzerland) was employed to perform the potentiostatic measurements. The counter electrode was a glassy carbon bar and a reversible hydrogen electrode (RHE) placed into a Luggin capillary acting as the reference electrode; all potentials presented in this manuscript are referred to this electrode. The working electrode was a glassy carbon disk modified with an ink of the Pd-Ni catalysts. This ink was prepared with 2.0 mg of catalyst, 15 μL of Nafion^®^ (5 wt %, Sigma-Aldrich S.L., Madrid, Spain) and 500 μL of ultrapure water; a 60 μL aliquot was deposited and dried on the glassy carbon disk. The currents presented in this work have been normalized by the electrochemical surface area, which was determined by the integration of the peak current area—bounded between 0.6 V and 0.7 V—detected during the backward scan, corresponding to the reduction of a PdO monolayer [75]. A charge of 405 mC·cm^−2^ involved in the reduction of this monolayer is considered. The solutions employed in the electrochemical test were prepared with high resistivity deoxygenated 18.2 MΩ H_2_O. For supporting electrolyte, 0.1 M KOH (99.99%, Sigma-Aldrich S.L., Madrid, Spain) was used and 1.0 M methanol (99%, Merck, Darmstadt, Germany) solutions were prepared for the methanol electrochemical characterization. CO strippings were made by bubbling CO (99.999%, Air Liquide, Zaragoza, Spain) into the three-electrode cell for 10 min. at 0.2 V vs. RHE, forming a CO monolayer on the catalyst surface; after, argon (99.999%, Air Liquide, Zaragoza, Spain) was bubbled for 20 min to remove the dissolved CO from the supporting electrolyte. Five potential scans at 20 mV·s^−1^ were performed, between 0.050 and 1.0 V. All the experiments were performed between 20 and 70 °C.

## 5. Conclusions

The synthesis of Pd-Ni catalysts supported on carbon nanofibers was performed, obtaining materials with metal contents close to 25 wt % and Pd:Ni atomic ratios near to 1:2. The carbon nanofibers allowed the obtaining of well-dispersed nanoparticles with few agglomerates, as was proven from the TEM images of these materials. The particle sizes were between 3 and 4 nm, diameters that probably promoted the oxidation of CO and methanol with low activation energies. In the case of the CO oxidation, the low E_act_ values were attributed to the existence of some crystallographic facets generated with the increase of the nanoparticle size, while in the case of the methanol oxidation, the structure of carbon nanofibers allowed the obtaining of high current densities. Moreover, the rate determining step during the methanol oxidation was the CO_ads_ diffusion toward the OH_ads_ for the materials supported on carbon nanofibers. The surface oxygen and nitrogen groups affected the particle size and, consequently, the CO oxidation E_act_.

## Figures and Tables

**Table 1:** Textural and chemical properties of carbon supports.

Carbon Supports	S_BET_/m^2^·g^−1^	V_total_/cm^3^·g^−1^	Nitrogen content/wt %
CNF	72.1	0.241	0.03
CNFO	72.7	0.260	0.13
CNFN	72.5	0.280	0.38
CB	214.6	0.412	----

**Table 2:** Physical characterization of Pd-Ni catalysts.

Catalyst	Atomic ratio Pd:Ni	Metal content/wt %	Particle size/nm
Pd-Ni/CNF 1:2	33:67	24	4.1 ± 1.2
Pd-Ni/CNFO 1:2	35:65	27	3.8 ± 1.2
Pd-Ni/CNFN 1:2	37:63	18	3.4 ± 1.1
Pd-Ni/CB 1:2	28:72	24	2.7 ± 0.9

**Table 3:** Electronic and composition parameters of the Pd-Ni catalysts supported on carbon nanofibers and carbon black, obtained from the X-ray photoelectron spectroscopy (XPS) analysis.

Catalyst	Pd			Ni		
	Binding Energy/eV	Relative Abundance/%	Assignment	Binding Energy/eV	Relative Abundance/%	Assignment
Pd-Ni/CNF 1:2 21:79 ^a^	335.7	34	Pd(II)	856.9	54	Ni(II)
	337.0	66		863.0	46	Ni(III)
							
Pd-Ni/CNFO 1:2 15:85 ^a^	336.0	62	Pd(II)	856.7	59	Ni(II)
	337.1	38		862.6	41	Ni(III)
							
Pd-Ni/CNFN 1:2 16:84 ^a^	335.9	60	Pd(II)	857.4	62	Ni(II)
	337.6	40		863.7	38	Ni(III)
							
Pd-Ni/CB 1:2 8:92 ^a^	336.2	93	Pd(II)	856.6	58	Ni(II)
	333.9	7	Pd (IV)	862.7	42	Ni(III)

^a^ Indicates the Pd:Ni atomic ratio on the surface determined from XPS analysis.

**Table 4:** CO oxidation activation energies for the synthesized Pd-Ni catalysts.

Catalyst	CO E_act_/kJ·mol^−1^
Pd-Ni/CNF 1:2	68
Pd-Ni/CNFO 1:2	101
Pd-Ni/CNFN 1:2	103
Pd-Ni/CB 1:2	118

**Table 5:** Methanol oxidation apparent activation energies for the synthesized Pd-Ni catalysts.

Catalyst	E_ap_/kJ·mol^−1^
Pd-Ni/CNF 1:2	15.2
Pd-Ni/CNFO 1:2	18.2
Pd-Ni/CNFN 1:2	30.3
Pd-Ni/CB 1:2	47.9
